# Electrode montage for transcranial direct current stimulation governs its effect on symptoms and functionality in schizophrenia

**DOI:** 10.3389/fpsyt.2023.1243859

**Published:** 2023-10-04

**Authors:** Yuji Yamada, Zui Narita, Takuma Inagawa, Yuma Yokoi, Naotsugu Hirabayashi, Aya Shirama, Kazuki Sueyoshi, Tomiki Sumiyoshi

**Affiliations:** ^1^Department of Psychiatry, National Center Hospital, National Center of Neurology and Psychiatry, Tokyo, Japan; ^2^Department of Behavioral Medicine, National Institute of Mental Health, National Center of Neurology and Psychiatry, Tokyo, Japan; ^3^Department of Educational Promotion, Clinical Research and Education Promotion Division, National Center of Neurology and Psychiatry, Tokyo, Japan; ^4^Department of Preventive Intervention for Psychiatric Disorders, National Institute of Mental Health, National Center of Neurology and Psychiatry, Tokyo, Japan

**Keywords:** neuromodulation, transcranial direct current stimulation (tDCS), schizophrenia, functional capacity, cognition

## Abstract

**Backgrounds:**

Patients with schizophrenia suffer from cognitive impairment that worsens real-world functional outcomes. We previously reported that multi-session transcranial direct current stimulation (tDCS) delivered to the left dorsolateral prefrontal cortex (DLPFC) improved daily living skills, while stimulation on the left superior temporal sulcus (STS) enhanced performance on a test of social cognition in these patients. To examine the region-dependent influence of tDCS on daily-living skills, neurocognition, and psychotic symptoms, this study compared effects of anodal stimulation targeting either of these two brain areas in patients with schizophrenia.

**Methods:**

Data were collected from open-label, single-arm trials with anodal electrodes placed over the left DLPFC (N = 28) or STS (N = 15). Daily-living skills, neurocognition, and psychotic symptoms were measured with the UCSD performance-based skills assessment-brief (UPSA-B), Brief Assessment of Cognition in Schizophrenia (BACS), and Positive and Negative Syndrome Scale (PANSS), respectively. After baseline evaluation, tDCS (2 mA × 20 min) were delivered two times per day for 5 consecutive days. One month after the final stimulation, clinical assessments were repeated.

**Results:**

Performance on the UPSA-B was significantly improved in patients who received anodal tDCS at the left DLPFC (*d* = 0.70, *p* < 0.001), while this effect was absent in patients with anodal electrodes placed on the left STS (*d* = 0.02, *p* = 0.939). Significant improvement was also observed for scores on the BACS with anodal tDCS delivered to the DLPFC (*d* = 0.49, *p* < 0.001); however, such neurocognitive enhancement was absent when the STS was stimulated (*d* = 0.05, *p* = 0.646). Both methods of anodal stimulation showed a significant improvement of General Psychopathology scores on the PANSS (DLPFC, *d* = 0.50, *p* = 0.027; STS, *d* = 0.44, *p* = 0.001).

**Conclusion:**

These results indicate the importance of selecting brain regions as a target for tDCS according to clinical features of individual patients. Anodal stimulation of the left DLPFC may be advantageous in improving higher level functional outcomes in patients with schizophrenia.

**Trial registration:**

These studies were registered within the University hospital Medical Information Network Clinical Trials Registry [(24), UMIN000015953], and the Japan Registry of Clinical Trials [(28), jRCTs032180026].

## 1. Introduction

Schizophrenia is one of the most prominent causes of disease burdens worldwide (1), with a prevalence of about 0.75% (2). The main symptoms of the disease include positive symptoms (e.g., hallucinations, delusions), negative symptoms (e.g., apathy, anhedonia, and social withdrawal), and disturbances of several types of cognitive function (e.g., neurocognition and social cognition). Positive symptoms are well treated with antipsychotic drugs, whereas negative symptoms and cognitive dysfunctions are not adequately managed by pharmacotherapy (3). In particular, cognitive impairment leads to a decline in real-world functional outcome, and more than 70% of chronic patients are not employed (4, 5).

Impairments of neurocognition and social cognition have been implicated in social functioning in patients with schizophrenia (6, 7). Neurocognitive domains, such as attention/processing speed, working memory, learning memory, and reasoning and problem solving, are most severely affected (7). On the other hand, social cognition domains affected in schizophrenia includes theory of mind (ToM), emotion recognition, social perception, and attributional bias (8, 9). Several interventional methods, e.g., psychosocial and pharmacological approaches, have been tested to enhance neurocognition and social cognition in patients with schizophrenia (10–16).

Transcranial direct current stimulation (tDCS) is a non-invasive brain stimulation method that modulates neural activity by applying electric currents, usually less than 2 mA, between an anode and cathode electrode for a short period of time (usually less than 30 min per session) (17). Previous meta-analyses reported that tDCS delivered to the dorsolateral prefrontal cortex (DLPFC) alleviates hallucinations (positive symptoms; Hedges’ g = 0.86) and negative symptoms (0.41), and improves neurocognitive function, particularly working memory (0.41), in patients with schizophrenia (18–23). Recently, tDCS targeting the DLPFC has also been reported to improve daily-living skills (functional capacity) (24), insight into the illness (25), and metacognition (26). Regarding social cognition, data from our systematic review indicate tDCS on the prefrontal cortex enhances emotion recognition (27), while stimulation on the left superior temporal sulcus (STS) improved scores on the ToM in these patients (28–30). Therefore, the electrode montage of tDCS, especially the anodal stimulation site, may determine its effect on symptoms and functionality in patients with psychotic conditions, although controversy exists (31–33). Taken together, further considerations are needed to understand which brain regions should be stimulated to improve specific symptoms of schizophrenia (34).

In the present study, we compared effects of anodal tDCS targeting the left DLPFC or left STS on symptoms and functional outcomes in patients with schizophrenia. For this purpose, data from our previous studies targeting either of the two brain regions (24, 28), with the same placement of *cathodal* electrodes and stimulus frequency and intensity, were analyzed. The previous study targeting the left DLPFC (24) measured three indicators, i.e., psychotic symptoms, neurocognition, and daily-living skills (functional capacity), except social cognition, whereas another previous study targeting the left STS, independently conducted (28), measured all four indicators including social cognition. The latter study did not report the results of daily-living skills (28). Therefore, the present study provides new information, i.e., the effect of anodal tDCS targeting the left STS on daily-living skills, as accumulated evidence suggests the performance on the functional capacity provides important indicator of social functioning (35).

## 2. Materials and methods

### 2.1. Participants and procedure

Data were collected from previous studies (Narita et al., UMIN000015953; Yamada et al., jRCTs032180026) (24, 28). The protocols of these studies and demographic data of participants have been reported (24, 28, 29). Both studies were open-label, single-arm trials, conducted in a single-center at the National Center of Neurology and Psychiatry, Japan. Twenty-eight patients with schizophrenia (22 inpatients and 6 outpatients) received anodal stimulation delivered to the left DLPFC (24), while 15 outpatients received the stimulation to the left STS (28). The former study about the anodal stimulation of the DLPFC demonstrated improvements in daily-living skills. Therefore, the latter study targeting the STS implemented a sample size calculation which was based on the results of daily-living skills. Specifically, the minimum number of samples to achieve a value of *p* of 0.05 and 80% power was found 13, assuming a standard deviation of 15 and a mean difference of 11. Taking drop-outs into account, the sample size was set at 15 (28). Both studies were independent and data were derived from non-controlled trials. Inclusion and exclusion criteria are provided in Supplementary Tables 1, 2. The details of data collection have been described (29).

### 2.2. Intervention

Direct current was transmitted through 35 cm^2^ saline-soaked sponge electrodes, and the intervention was performed by a 1 × 1 transcranial direct current low-intensity stimulator (Model 1,300 A; Soterix Medical Inc., New York, NY, United States). For tDCS montage, the anode was placed on F3 (the international 10–20 electroencephalography system) to deliver currents to the left DLPFC or T3 to the left STS, while the cathode was placed on FP2 regions in both cases. We applied 10 sessions direct current of 2 mA for 20 min on 5 consecutive days (twice per day, with an interval of 30 min; Supplementary Table 3).

### 2.3. Outcomes

After being briefed on the purpose of the study and agreeing to participate, patients received psychological and clinical assessments, including the screening evaluation. Data were collected at baseline and 1 month after the final stimulus (Supplementary Table 3).

#### 2.3.1. Daily-living skills (functional capacity)

Daily-living skills were assessed by the UCSD Performance-Based Skills Assessment-Brief (UPSA-B), which consists of financial and communication skills (36). Subscale scores of the two domains of the UPSA-B (i.e., finances and communication) were converted into the standard score ranging from 0 to 50 to make the maximum of the total score of 100. Higher scores indicate greater functional capacity.

#### 2.3.2. Neurocognition

The Brief Assessment of Cognition in Schizophrenia (BACS) Japanese version was used to evaluate verbal memory (verbal memory list learning), working memory (digit sequencing task), speed of information processing (symbol coding), motor speed (token motor task), executive functions (tower of London) and verbal fluency (37). To provide a standard metric for combining test scores into domains and comparing performance over time, BACS scores were converted to z-scores to represent performance relative to that of healthy people (38).

#### 2.3.3. Psychotic symptoms

The Positive and Negative Syndrome Scale (PANSS) was used to assess psychotic symptoms (39). The PANSS was a structured interview, consisting of positive, negative, and general psychopathology subscales (with scores ranging from 7 to 49, from 7 to 49, and from 16 to 112, respectively). The higher scores represent more severe psychotic symptoms.

### 2.4. Statistical analysis

Student’s *t*-test was used for the clinical outcomes to evaluate the efficacy, while effect sizes were calculated as standardized mean difference (Cohen’s d). Categorical variables were compared by the Chi-Squared test. Statistical analysis was conducted using IBM SPSS Statistics version 26.0.

## 3. Results

### 3.1. Participants

The demographic variables are shown in Table 1. There were no differences between participants in the two studies in potential parameters affecting cognitive/functional outcomes, i.e., age, duration of present illness, duration of education, estimated premorbid Intelligence Quotient (IQ), dose of antipsychotics, and *severity of psychotic symptoms*. No medication was changed, nor was cognitive rehabilitation performed during the study period.

**Table 1:** Clinical characteristics of patients [Narita et al. (24), Yamada et al. (28)].

Anodal stimulation sites	Left DLPFC	Left STS	
Variables	Mean (SD) or *n*	Mean (SD) or *n*	*p* Value
Inpatient/outpatient	22/6	0/15	**<0.001**
Male/female	16/12	7/8	0.510
Age (year)	40.9 (9.8)	40.1 (11.8)	0.813
Duration of present illness (year)	17.3 (9.9)	12.6 (10.2)	0.149
Duration of education (year)	13.8 (1.7)	13.6 (1.8)	0.720
Premorbid IQ	99.6 (12.0)	102.1 (11.0)	0.506
Chlorpromazine equivalent dose of antipsychotics (mg/day)	889.0 (587.1)	727.9 (323.1)	0.331
PANSS (positive syndrome)	15.7 (5.7)	16.2 (6.0)	0.792
PANSS (negative syndrome)	14.9 (8.0)	19.3 (6.4)	0.057
PANSS (general psychopathology)	32 (8.1)	37.5 (9.2)	0.062

DLPFC, dorsolateral prefrontal cortex; STS, superior temporal sulcus; SD, standard deviation; IQ, Intelligence Quotient; PANSS, Positive and Negative Syndrome Scale. Values reaching statistical significance are bolded.

### 3.2. Daily-living skills (functional capacity)

Performance on the UPSA-B was significantly improved in patients who received anodal tDCS at the left DLPFC (*d* = 0.70, *p* < 0.001), while this advantage was absent with anodal electrodes placed on the STS (*d* = 0.02, *p* = 0.939; Table 2). Similarly, anodal stimulation on the DLPFC significantly improved scores on the tests of financial skills (*d* = 0.61, *p* = 0.002) or communication skills (*d* = 0.59, *p* = 0.001) from the UPSA-B, while such changes were absent in patients with anodal electrodes for the STS (financial skills, *d* = 0.14, *p* = 0.635; communication skills, *d* = 0.07, *p* = 0.760; Table 2).

**Table 2:** Outcome measures at baseline and 1 month after the transcranial direct current stimulation (tDCS).

Anodal stimulation sites	Left DLPFC					Left STS				
	Baseline, mean (SD)	Follow-up, mean (SD)	*t* Value (Degree of freedom)	*p* Value	Effect size	Baseline, mean (SD)	Follow-up, mean (SD)	*t* Value (Degree of freedom)	*p* Value	Effect size
UPSA-B										
Total	68.4 (14.8)	79.0 (15.5)	*t* = 5.89 (27)	**<0.001**	***d* = 0.70**	72.7 (10.3)	72.5 (16.0)	*t* = 0.07 (14)	0.939	*d* = 0.02
Financial skills	41.4 (8.1)	45.8 (6.2)	*t* = 3.35 (27)	**0.002**	***d* = 0.61**	45.3 (4.4)	44.4 (7.3)	*t* = 0.48 (14)	0.635	*d* = 0.14
Communication skills	27.1 (9.6)	33.2 (11.1)	*t* = 3.57 (27)	**0.001**	***d* = 0.59**	27.4 (9.2)	28.0 (10.3)	*t* = −0.31 (14)	0.760	*d* = 0.07
BACS (z-score)										
Composite score	−1.86 (0.92)	−1.40 (0.93)	*t* = 4.23 (27)	**<0.001**	***d* = 0.49**	−1.85 (1.36)	−1.79 (1.29)	*t* = −0.46 (14)	0.646	*d* = 0.05
Verbal memory	−1.67 (1.06)	−1.06 (1.14)	*t* = 4.53 (27)	**<0.001**	***d* = 0.55**	−1.00 (1.17)	−0.99 (1.15)	*t* = −0.09 (14)	0.929	*d* = 0.01
Working memory	−1.16 (1.38)	−0.95 (1.37)	*t* = 1.52 (27)	0.14	*d* = 0.15	−1.02 (1.18)	−0.90 (1.06)	*t* = −0.06 (14)	0.513	*d* = 0.11
Motor speed	−3.27 (1.25)	−2.73 (1.23)	*t* = 2.47 (27)	**0.020**	***d* = 0.44**	−1.80 (0.93)	−1.81 (1.17)	*t* = 0.04 (14)	0.965	*d* = 0.01
Verbal fluency	−1.19 (1.05)	−0.84 (0.89)	*t* = 2.10 (27)	**0.046**	***d* = 0.36**	−0.85 (0.97)	−0.66 (0.81)	*t* = −1.48 (14)	0.159	*d* = 0.21
Attention and speed	−2.25 (1.22)	−2.21 (1.44)	*t* = 0.25 (27)	0.80	*d* = 0.03	−1.35 (0.88)	−1.10 (0.95)	*t* = −2.10 (14)	0.054	*d* = 0.27
Executive functions	−1.76 (2.03)	−1.12 (2.16)	*t* = 1.88 (27)	0.071	*d* = 0.31	−0.18 (1.41)	−0.56 (1.20)	*t* = 1.54 (14)	0.143	*d* = 0.29
PANSS										
Positive syndrome	15.7 (5.7)	13.1 (4.8)	*t* = 2.31 (27)	**0.029**	***d* = 0.48**	16.2 (6.0)	15.6 (6.3)	*t* = 0.78 (14)	0.444	*d* = 0.10
Negative syndrome	14.9 (8.0)	13.6 (6.7)	*t* = 1.24 (27)	0.23	*d* = 0.17	19.3 (6.4)	17.9 (6.2)	*t* = 1.56 (14)	0.139	*d* = 0.22
General psychopathology	32 (8.1)	28.3 (7.1)	*t* = 2.35 (27)	**0.027**	***d* = 0.50**	37.5 (9.2)	33.4 (9.2)	*t* = 4.07 (14)	**0.001**	***d* = 0.44**

tDCS, transcranial direct current stimulation; DLPFC, dorsolateral prefrontal cortex; STS, superior temporal sulcus; SD, standard deviation; UPSA-B, the UCSD performance-based skills assessment-brief; BACS, Brief Assessment of Cognition in Schizophrenia; PANSS, Positive and Negative Syndrome Scale. Values reaching statistical significance are bolded.

### 3.3. Neurocognition

Significant improvement was observed on the composite score of the BACS in patients who received anodal tDCS delivered to the left DLPFC (*d* = 0.49, *p* < 0.001), while such neurocognitive enhancement was absent with anodal stimulation targeting the left STS (*d* = 0.05, *p* = 0.646; Table 2). Similarly, anodal stimulation delivered to the DLPFC was associated with improvements in verbal memory (*d* = 0.55, *p* < 0.001), motor speed (*d* = 0.44, *p* = 0.020), and verbal fluency (*d* = 0.36, *p* = 0.046), while these benefits were absent when anodal stimulation was targeted to the STS (verbal memory, *d* = 0.01, *p* = 0.929; motor speed, *d* = 0.01, *p* = 0.965; verbal fluency, *d* = 0.21, *p* = 0.159; Table 2).

### 3.4. Psychotic symptoms

Anodal tDCS to either the left DLPFC (*d* = 0.50, *p* = 0.027) or STS (*d* = 0.44, *p* = 0.001) showed a significant improvement of general psychopathology scores on the PANSS. On the other hand, anodal stimulation delivered to the DLPFC (*d* = 0.48, *p* = 0.029), but not the STS (*d* = 0.10, *p* = 0.444) was associated with amelioration of positive symptoms. For negative symptoms, neither stimulation method was effective (DLPFC, *d* = 0.17, *p* = 0.230; STS, *d* = 0.22, *p* = 0.139).

## 4. Discussion

The results of the present study indicate that the placement of electrodes influences the ability of tDCS to ameliorate symptoms and improve functional outcome in patients with schizophrenia. This concept is supported by observations that anodal stimulation delivered to the left DLPFC, but not left STS was efficacious in enhancing daily-living skills and alleviating positive symptoms, while stimulation of the latter brain region was associated with improvement of social cognition.

Several neural substrates may explain the ability of anodal tDCS to enhance cognitive function and related functional capacity (daily-living skills). Altered structures in the brain of patients with schizophrenia has been reported, e.g., reduced grey matter in the frontotemporal lobe, thalamus, hippocampus, amygdala, insular cortex, and anterior cingulate cortex (40). Specifically, neurocognitive dysfunction is assumed to result from atrophy of the hippocampus and the DLPFC (40). The degree of change in brain morphology varies among individuals with schizophrenia, yielding variances of electric fields in brain cortical areas produced by tDCS with the same electrode montage (31–33).

Data from the present study indicate differential clinical benefits which depends on the placement of the anodal electrode. Traditionally, social cognitive function has been associated with the neural circuitry involving the STS, medial prefrontal cortex, and middle temporal gyrus (29). Despite previous observations (7, 9, 41, 42), the results of the present study suggest that social cognition does not appear to be directly linked to improvements in functional capacity, as indicated by the lack of efficacy anodal tDCS on the STS (28). In sum, the ability of anodal stimulation on the DLPFC, but not STS to enhance functional capacity (daily-living skills) provides insight into neural mechanisms for therapeutics to improve higher functional outcomes in patients with schizophrenia.

The amelioration of positive symptoms by anodal tDCS delivered to the left DLPFC indicates the role for this brain region in the treatment of psychotic conditions. Specifically, the neural circuit with the hippocampus as a hub may play a role. Thus, anodal stimulation on the DLPFC is assumed to modulate the activities of hippocampus through this neural circuit, possibly via alterations of monoaminergic neurotransmissions (17). This concept is consistent with observations that increased activities of the hippocampus are related to positive symptoms (40). Therefore, the ability of anodal stimulation on the DLPFC to alleviate positive symptoms may be related to modulation of hyperactivity of the hippocampus.

Data from the current study suggest the importance of selecting brain areas to be targeted by tDCS according to clinical features of individual patients. The frontal brain regions, e.g., the left DLPFC, has been used in most studies showing improvement of psychotic symptoms and functionality in patients with schizophrenia (18, 19, 24), although some argued against this concept (31–33, 43, 44). However, these brain areas may not be ideal as a target for alleviating social cognitive impairment (27, 29, 30). Further study is warranted to confirm the present results with randomized controlled trial with target brain regions of the DLPFC or STS.

The limitations of this study should be mentioned. First, the small sample size may raise caution in generalizing the present results. Second, as we compared results from previous studies with different populations, e.g., the ratio of inpatient/outpatient number across the two studies, it cannot be ruled out that the characteristics of participants may have influenced the results. The modest internal validity and low external validity indicate the need for further validation before generalizing the present findings to clinical practice.

## 5. Conclusion

The results of the present study suggest that anodal stimulation site of tDCS may govern its effect on symptoms and functionality in patients with schizophrenia. For improving daily-living skills associated with neurocognition, the left DLPFC may provide an optimal brain region to be targeted.

## Data availability statement

The original contributions presented in the study are included in the article/Supplementary material, further inquiries can be directed to the corresponding author.

## Ethics statement

The studies involving humans were approved by the National Center of Neurology and Psychiatry Clinical Research Review Board (CRB3180006). The studies were conducted in accordance with the local legislation and institutional requirements. The participants provided their written informed consent to participate in this study.

## Author contributions

YYa and TS developed the original concept for the study and designed it. ZN advised on the statistical analysis, while KS advised on the outcome measures. AS, YYa, and ZN administered tDCS. TI and TS recruited participants. TS organized the team for this study. YYa wrote the original draft. All other authors reviewed and commented on the subsequent drafts, and approved the submitted version.
