# Supplementary material for: Electrode montage for transcranial direct current stimulation governs its effect on symptoms and functionality in schizophrenia
Source: Front Psychiatry. 2023 Oct 4;14:1243859. doi: 10.3389/fpsyt.2023.1243859 (PMC10582326; doi:10.3389/fpsyt.2023.1243859)
Supplement: Supplementary file 1 [file Data_Sheet_1.pdf]

Supplementary Table 1. Inclusion and exclusion criteria (Narita et al. (2017) [24]).

	Inclusion criteria
1	Meeting DSM-5 criteria for schizophrenia.
2	Being 20 through 60 years old.
3	Being able to sign and give consent.
	Exclusion criteria
1	Alcohol or substance disorder
2	Traumatic brain injury
3	Epilepsy
	Recruitment methods
	Inpatients or outpatients treated at National Center Hospital, National Center of Neurology and Psychiatry, were enrolled. Participants were recruited by psychiatrists' referrals. They provided written informed consent before starting the trial.

Supplementary Table 2. Inclusion and exclusion criteria (Yamada et al. (2021) [29]).

	Inclusion criteria
1	Diagnosis of schizophrenia in DSM-5 was made by well trained and experienced clinicians with extensive clinical research experience in the National Center of Neurology and Psychiatry.
2	Aged between 20 and 70.
3	Being able to understand the objectives and content of the study, and provide consent to participate in it. (The ability to consent to participate in this study is considered insufficient, when patients' Intelligence Quotient (IQ) is less than 70, or they present with acute psychiatric symptoms. Those patients are provided with necessary medical care separately from this trial.)
4	Having Social Cognition Screening Questionnaire (SCSQ) scores of less than 34 points.
	Exclusion criteria
1	Present or past history of severe organic lesions in the brain, dementia, or epilepsy.
2	With alcohol or substance use disorder that was present within 12 months from screening.
3	Contraindicated against electro convulsive therapy or tDCS, e.g., severe cardiovascular diseases, such as myocardial infarction, or aneurysms at high risk of rupture.
4	Were treated with tDCS or other neuromodulations within the past 2 months. (We asked whether participants had any history of tDCS or other neuromodulations.)
5	Deemed inappropriate to participate judged by the principal investigator, e.g., when participants' psychiatric symptoms were unstable.
	Recruitment methods
	Inpatients or outpatients treated at National Center Hospital, National Center of Neurology and Psychiatry were enrolled. Participants were recruited by referrals from treating psychiatrists. Those psychiatrists did not have any conflicts of interest with the outcomes of this trial. The principal investigator must provide written informed consent before starting the trial. After providing the informed consent, participants were screened by a treating psychiatrist to establish whether they met the eligibility criteria.

Supplementary Table 3-1. Study schedules (Narita et al. (2017) [24]).

Title	Study Period		
	Baseline	Intervention	Follow-up
**Time point**	Week 1	Week 2	Week 7
**Enrollment**			
Eligibility screen	X		
Informed consent	X		
Sociodemographic characteristics	X		
**Intervention**			
tDCS (twice/day)		←→	
**Assessments**			
UPSA-B	X		X
BACS	X		X
PANSS	X		X
Adverse events	X	←→	X

tDCS, transcranial direct current stimulation; UPSA-B, the UCSD performance-based skills assessment-brief; BACS, Brief Assessment of Cognition in Schizophrenia; PANSS, Positive and Negative Syndrome Scale.

Supplementary Table 3-2. Study schedules (Yamada et al. (2021) [29]).

Title	Study Period				
	Baseline	Intervention			Follow-up
**Time point**	Within 2 weeks before the start of intervention	Day 1	Days 2-4	Day 5	1 month after the end of the last stimulation
**Enrollment**					
Eligibility screen	X				
Informed consent	X				
Sociodemographic characteristics	X				
**Intervention**					
tDCS (twice/day)		X	X	X	
**Assessments**					
UPSA-B	X				X
BACS	X				X
PANSS	X				X
Adverse events	X	X	X	X	X
Prescribed drugs	X	X	X	X	X

tDCS, transcranial direct current stimulation; UPSA-B, the UCSD performance-based skills assessment-brief; BACS, Brief Assessment of Cognition in Schizophrenia; PANSS, Positive and Negative Syndrome Scale.
